# Therapeutic Potential of Thiosemicarbazone Derivative in LPS‐Induced Mastitis: Dual Modulation of Inflammatory Response and Blood–Milk Barrier Integrity

**DOI:** 10.1155/jimr/7518397

**Published:** 2026-09-21

**Authors:** Ling Guo, Yining Wang, Jia Liang, Ying Liu, Wei Tao

**Affiliations:** ^1^ Institution of Life Science, Jinzhou Medical University, Jinzhou, China, jzmu.edu.cn; ^2^ Department of Cardiovascular Surgery, Jinzhou Central Hospital, Jinzhou, China; ^3^ The First Affiliated Hospital of Jinzhou Medical University, Jinzhou, China, jzmu.edu.cn; ^4^ Key Laboratory of Neurodegenerative Diseases of Liaoning Province and Department of Neurobiology, Jinzhou Medical University, Jinzhou, China, jzmu.edu.cn; ^5^ Institution of Basic Medical Science, Jinzhou Medical University, Jinzhou, China, jzmu.edu.cn

**Keywords:** blood–milk barrier, inflammation, mastitis, NF-κB pathway, thiosemicarbazone derivative

## Abstract

Mastitis is a prevalent inflammatory disorder in women, this condition exhibits a particularly elevated prevalence among breastfeeding women, which significantly compromises mammary health and lactation function. This research explored the anti‐inflammatory properties of YWJ‐6 in lipopolysaccharide (LPS)‐induced mastitis, its protective role on the blood–milk barrier (BMB), and the underlying mechanisms. An LPS‐induced mastitis rat model was established. H&E staining assessed mammary tissue damage, and myeloperoxidase (MPO) activity was measured. RT‐qPCR and IHC were used to demonstrate interleukin 1β (IL‐1β), interleukin 6 (IL‐6), and tumor necrosis factor alpha (TNFα) expression. These data indicated that inflammatory injury was alleviated. Following drug treatment, MPO expression and the mRNA/protein expression of these inflammatory factors were suppressed. Immunofluorescence staining analyzed the distribution of Zonula Occludens‐1 (ZO‐1), Occludin, and Claudin‐3. WB quantitatively detected ZO‐1 and Occludin expression, confirming that YWJ‐6 promoted tight junction (TJ) protein expression restoration and distribution integrity, repairing the damaged BMB. Consistently, Evans blue extravasation assay demonstrated that YWJ‐6 reduced mammary vascular permeability, providing direct functional evidence for BMB protection. mIHC detected the colocalization of CD68, MPO, and nuclear factor‐kappa B (NF‐κB) p65 subunit (NF‐κB p65), showing that YWJ‐6 modulated inflammatory cell infiltration and p65 activation. RT‐qPCR, WB, and ELISA assays in HC11 and RAW264.7 cells confirmed that YWJ‐6 downregulated inducible nitric oxide synthase (iNOS), cyclooxygenase‐2 (COX‐2) and IL‐1β, IL‐6 and TNF‐α expression in HC11 cells, and suppressed agent responsible for inflammation secretion in RAW264.7 cells. WB and immunofluorescence showed that YWJ‐6 inhibited inhibitor of NF‐κB alpha (IκBα) and NF‐κB p65 phosphorylation in both HC11 and RAW264.7 cells. In conclusion, YWJ‐6 exerts dual protective effects by targeting NF‐κB signaling pathway activation, providing experimental evidence for mastitis treatment in future.

## 1. Introduction

Mastitis represents a typical benign inflammation of the breast tissue [1–3]. The incidence of mastitis has shown an increasing trend. Mastitis includes lactational and nonlactational mastitis [4]. Epidemiological studies suggest that mastitis, particularly chronic or recurrent mastitis, is associated with an elevated risk of breast carcinogenesis [5]. Lactational mastitis, the most common inflammatory condition among postpartum women, typically occurs within 6–8 weeks after delivery, with its incidence ranging from 3% to 33% due to regional and cultural variations [4]. Based on the disease duration, lactational mastitis can be further categorized into acute and subacute mastitis [6]. Acute mastitis presents distinct clinical features and is easily diagnosable, thus being the most prevalent subtype during lactation [6]. The vast majority of pathogens causing lactation mastitis are staphylococci [7]. Milk stasis creates a favorable environment for bacterial proliferation, enabling pathogens such as *Staphylococcus aureus* to ascend into the mammary ducts and trigger an acute inflammatory response in the mammary tissue [8]. Clinical manifestations of lactational mastitis include breast redness, swelling, pain, and fever [9]. Without timely intervention, complications such as breast abscess may occur, which can hinder successful breastfeeding [10, 11]. In pharmacological management, antibiotics play a crucial role; however, their overuse poses risks, including drug resistance, hepatotoxicity, nephrotoxicity, and intestinal dysbiosis. Exploring safe and effective alternatives to antibiotics is therefore of paramount importance for improving treatment outcomes.

YWJ‐6 is a thiosemicarbazone derivative. Thiosemicarbazone derivatives have garnered considerable attention in drug discovery and development [12]. In multiple diseases, such compounds can bind to residual sites to influence disease progression [13, 14]. Thiosemicarbazone derivatives are structurally derived from the process of amines reacting with aldehydes or ketones via condensation. The presence of the C═N double bond and surrounding functional groups facilitate specific interactions with a diverse array of biomolecules, including enzymes, receptors, and nucleic acids, thereby conferring potent biological activities [15]. Numerous studies have reported that thiosemicarbazone derivatives exhibit antigerm, anti‐inflammation, antioxidizing, and antineoplastic effects [16]. Regarding their anti‐inflammatory properties, thiosemicarbazone derivatives have been shown to mitigate the inflammatory response by altering pathways such as the nuclear factor‐kappa B (NF‐κB) pathway [17]. However, while its antitumor effects have been studied in the literature as a newly synthesized thiocysteine derivative [18], there is currently very little research on the effects of YWJ‐6 on mastitis.

*Escherichia coli* (*E. coli*) ranks among the most common pathogens causing intense mastitis [19]. LPS entering the mammary tissue can induce a strong inflammatory response and excessive production of inflammatory mediators, which subsequently damages tissues [20–23]. What is more, LPS disrupts the integrity of the blood–milk barrier (BMB) [24]. However, the effect of YWJ‐6 on mastitis is still not well understood. Therefore, this research aims to observe the regulatory impacts of YWJ‐6 on mastitis. The results provided experimental support for the possibility of application of YWJ‐6 in mastitis therapy.

## 2. Materials and Methods

### 2.1. Animals

Female Sprague‐Dawley rats (*n* = 30, 250–280 g) were obtained from the Experimental Animal Center of Jinzhou Medical University. Pregnant animals were housed under controlled conditions (24 ± 2°C, 50 ± 5% humidity, and 12 h light/dark cycle) with free access to food and water. All procedures were approved by the Animal Welfare Protection and Utilization Committee of Jinzhou Medical University.

In the intraperitoneal administration experiment, five subgroups of pregnant female rats at 2 weeks gestation (*n* = 6 per group) were established: a normal control group (Control), a lipopolysaccharide (LPS)‐induced mastitis group, and three treatment groups with different drug concentrations. YWJ‐6 was fully characterized by ^1^H‐NMR, ^13^C‐NMR, HR‐MS, and HPLC, as detailed in Figures S1–S4. In the YWJ‐6+LPS group, the specific details were as follows: the low, medium, and high drug concentrations correspond to 2.5 mg/kg, 5 mg/kg, and 10 mg/kg, respectively [25]. The model for mastitis was created through the intramammary injection of LPS on postpartum days 3–5. Rats in the LPS + YWJ‐6‐L, LPS + YWJ‐6‐M, and LPS + YWJ‐6‐H groups received intraperitoneal injections of YWJ‐6 (0.5 mL) once at 16 and 2 h before mastitis induction and again at 6 h after induction. About 0.5 mL of PBS was administered to both Control group and LPS group rats using the identical method. The administration method was based on the relevant literature [26].

### 2.2. Construction of the Mastitis Model

First, female rats were isolated from their offspring for 2 h before establishing the model. The animals were put under anesthesia via the inhalation of 2%–3% isoflurane. After abdominal hair removal treatment, the sample was aseptically disinfected with ethanol at a concentration of 75%. Next, a 50 μL volume of LPS solution (0.8 μg/μL, *E. coli* O55: B5, Sigma–Aldrich, USA) was slowly injected into the mammary ducts using a micro syringe. For the Control group, 50 μL of PBS was substituted. After 24 h, following cervical dislocation euthanasia, mammary tissues were gathered from the rats.

### 2.3. H&E Staining

Mammary tissues were fixed in 4% paraformaldehyde for 48 h, embedded in paraffin, and sectioned at 4 μm. Sections were stained with hematoxylin and eosin for histopathological examination.

### 2.4. Myeloperoxidase (MPO) Assay

The collected breast tissue was placed in an EP tube containing PBS for grinding. After thorough grinding under low‐temperature conditions, the supernatant was obtained by centrifugation. MPO levels were assessed via ELISA.

### 2.5. IHC Staining

Paraffin sections were deparaffinized, rehydrated, and subjected to antigen retrieval in Tris‐EDTA buffer. After blocking with 5% BSA, sections were incubated overnight at 4°C with primary antibodies against interleukin 1β (IL‐1β) (1:400), interleukin 6 (IL‐6) (1:300), and TNF‐α (1:600) (Abcam, USA), followed by HRP‐conjugated secondary antibodies. Stained sections were imaged and quantified using ImageJ software.

### 2.6. mIHC Staining

Multiplex immunofluorescence staining was performed using the Opal 7‐Color Automation Kit (Akoya, USA). Sections were sequentially stained with primary antibodies against CD68 (1:300, Cell Signaling Technology, Shanghai, China), MPO (1:200, Proteintech, Wuhan, China), and p65 (1:50, Absin, Shanghai, China), followed by HRP polymer and tyramine signal amplification (Opal 520/570/620). DAPI was used for nuclear counterstaining. Images were acquired using the Mantra Tissue Section Quantitative Analysis System (Leica, Germany) and analyzed with the inForm 2.6.0 software.

### 2.7. Evans Blue Extravasation Assay

To assess the permeability of the BMB) in vivo, Evans Blue dye (a 2% solution in PBS, 4 mL/kg body weight) was administered via the tail vein 1 h before euthanasia. Mammary gland tissue was harvested, weighed, and incubated in trichloroacetic acid at 4°C for 24 h to extract the dye. The absorbance of the supernatant was measured at 620 nm, and the Evans Blue concentration was calculated based on a standard curve; the results are expressed in μg/100 mg of tissue.

### 2.8. Cultivation and Processing of Cells

The HC11 mouse mammary epithelial cell line and RAW264.7 murine monocyte–macrophage cell line were purchased from Procell Life Science & Technology Co., Ltd. RAW264.7 cells were cultured in DMEM (Gibco, USA) with 10% fetal bovine serum (FBS) (AusgeneX, Australia), 1% penicillin–streptomycin (Pen‐Strep) (Gibco, USA), and 1% non‐essential amino acids (MEM NEAA; Gibco, USA) at 37°C in a 5% CO_2_. HC11 cells were cultivated in a HC11‐specific medium (Procell, Wuhan, China) under the same conditions. For LPS stimulation, cells were pretreated with YWJ‐6 (0.3125–5 μmol/L) for 2 h, followed by LPS (1 μg/mL for RAW264.7 and 5 μg/mL for HC11) for 18 h. Cell supernatants and adherent cells were gathered for further experiments.

### 2.9. Survival Capacity Assessment for Cells

HC11 (4 × 10^3^) and RAW264.7 (8 × 10^3^) cells were placed in 96‐well plates, respectively. After a night had passed, the cells were assigned to the following groups at random: Control and YWJ‐6 (0.3125, 0.625, 1.25, 2.5, and 5 μmol/L). After the supernatant was discarded, 0.2 mL of medium without serum supplemented with CCK‐8 (Beyotime Biotechnology, Shanghai, China) was added to all wells and fostered for 60 min. Absorbance (*A*_450_) was obtained using a multifunctional microplate reader (BioTek, USA).

### 2.10. Lactate Dehydrogenase (LDH) Release Assay

The effect of YWJ‐6 on LDH release from LPS‐stimulated HC11 cells was detected. HC11 cells (2 × 10^3^) were placed in 96‐well plates. Stimulation of HC11 cells with LPS was prolonged to 48 h. A portion of the supernatant was aspirated and reacted with the LDH assay kit (Beyotime Biotechnology, Shanghai, China). The absorbance at 490 nm was read via a multifunctional microplate reader.

### 2.11. RNA Extraction and RT‐qPCR

Total RNA was purified from mammary tissue and cultured cells utilizing the Trizol reagent (Solarbio, Beijing, China). RNA concentration and purity were determined using a Nanodrop spectrophotometer. cDNA was synthesized using a Reverse Transcription Kit (Thermo Fisher Scientific, USA). Real‐time PCR was performed using the SYBR Green premix (Thermo Fisher Scientific, USA) with specific primers. Relative gene expression was calculated using the 2^−ΔΔCt^ method with β‐actin as the internal control. The primers employed in the assay are shown in Table 1.

**Table 1:** Rat primer sequences.

Gene	Primers sequence (5′→3′)
IL‐1β	F:CAGCAGCATCTCGACAAGAG
	R:AAAGAAGGTGCTTGGGTCCT
IL‐6	F:TCTATACCACTTCACAAGTCGGA
	R:GAATTGCCATTGCACAACTCTTT
TNF‐α	F:GGAGGGAGAACAGCAACTCC
	R:GCCAGTGTATGAGAGGGACG
β‐actin	F:ACATCCGTAAAGACCTCTATGCC
	R:TACTCCTGCTTGCTGATCCAC

RNA extraction from cells follows the same procedure as that for tissue extraction. All primer employed are detailed in Table 2.

**Table 2:** Mouse primer sequences.

Gene	Primers sequence (5′→3′)
IL‐1β	F:TGAAATGCCACCTTTTGACAG
	R:CCACAGCCACAATGAGTGATAC
IL‐6	F:TGCCTTCTTGGGACTGAT
	R:CTGGCTTTGTCTTTCTTGTT
TNF‐α	F:CGATGAGGTCAATCTGCCCA
	R:CCAGGTCACTGTCCCAGC
β‐actin	F:TGCTGTCCCTGTATGCCTCT
	R:GGTCTTTACGGATGTCAACG

### 2.12. Elisa Assay

The ELISA kits (Youke Life Biotechnology, Hangzhou, China) were used to detect IL‐6 and TNF‐α secretion in RAW264.7 cells. The supernatant was collected and processed according to the kits’ operating procedures.

### 2.13. Immunofluorescence Assay

Tissue sections or cell slides were incubated in 4% paraformaldehyde, permeabilized with 0.1% Triton X‐100, and blocked with 5% donkey serum. Samples were incubated overnight at 4°C with primary antibodies against Zonula Occludens‐1 (ZO‐1) (1:100, Proteintech, Wuhan, China), Occludin (1:100, ZENBIO, Sichuan, China), Claudin‐3 (1:200, MedChemExpress, USA), or phospho‐p65 (1:100, Cell Signaling Technology, Shanghai, China). After washing, samples were incubated with FITC/RBITC‐conjugated secondary antibodies (1:200, Solarbio, Beijing, China) for 1 h at room temperature in the dark. The nuclei were stained with DAPI (1:100, 5 min). Images were acquired using a fluorescence microscope (Leica, Germany).

### 2.14. Western Blot Assay

Total proteins were extracted from mammary tissue, RAW264.7, and HC11 cells using RIPA lysis buffer containing PMSF and a protein phosphatase inhibitor. Protein samples were separated by SDS–PAGE, transferred to PVDF membranes, and blocked with 5% nonfat milk. Membranes were incubated overnight at 4°C with primary antibodies against cyclooxygenase‐2 (COX‐2) (1:1000, Proteintech), inducible nitric oxide synthase (iNOS) (1:500, Wanlei), ZO‐1 (1:5000), Occludin (1:1000), p65 (1:1000), phospho‐p65 (1:1000), inhibitor of NF‐κB alpha (IκBα) (1:1000), phospho‐IκBα (1:800), and β‐actin (1:3000). After incubation with secondary antibodies, protein bands were visualized using the Odyssey CLx system (LI‐COR) and quantified with Image Studio software. Protein expression was normalized to β‐actin. Western blot images are shown in Figure S5.

### 2.15. Statistical Analysis

The data are shown as the means ± standard error (SEM). Comparisons between two groups were performed using Student’s *t*‐test, while comparisons among three or more groups were performed using one‐way analysis of variance (ANOVA). *p*  < 0.05 was deemed statistically significant.

## 3. Results

### 3.1. YWJ‐6 Attenuated LPS‐Induced Mammary Gland Injury in Rats

**Figure 1:**
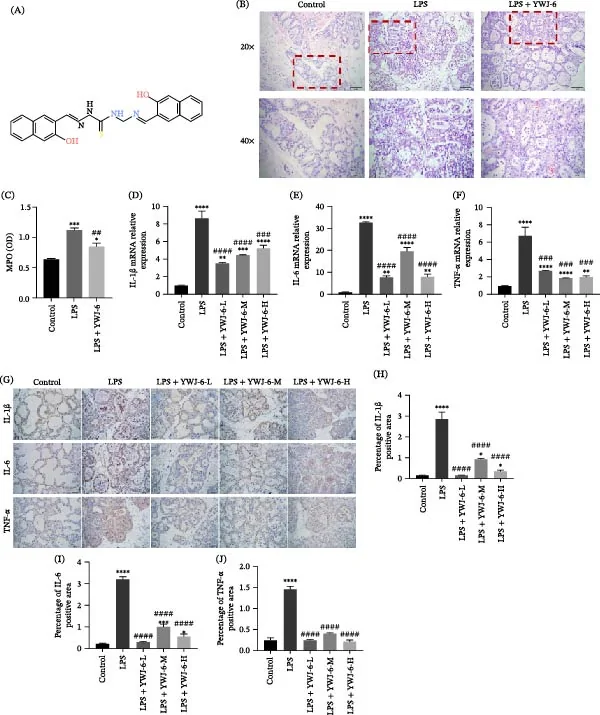
YWJ‐6 attenuates LPS‐induced mastitis in rats. (A) Chemical structure of YWJ‐6. (B) H&E staining of mammary gland tissues from Control, LPS, and LPS + YWJ‐6 groups. (C) MPO expression in mammary gland tissues. (D–F) mRNA expressions levels of IL‐β, IL‐6, and TNF‐α in mammary gland tissues were measured using RT‐qPCR. (G) Representative IHC staining of IL‐β, IL‐6, and TNF‐α in mammary gland tissues. (H–J) Quantitative analysis of IHC positive area fraction for IL‑1β, IL‑6 and TNF‑α in mammary gland tissues.  ^∗^ *p* < 0.05,  ^∗∗^ *p* < 0.01,  ^∗∗∗^ *p* < 0.001, and  ^∗∗∗∗^ *p* < 0.0001 versus Control group; ##*p* < 0.01, ###*p* < 0.001, and ####*p* < 0.0001 versus LPS group. Figure B 20x scale = 100 μm, Figure B 40x, and Figure G scale = 50 μm (*n* = 3).

**Figure 2:**
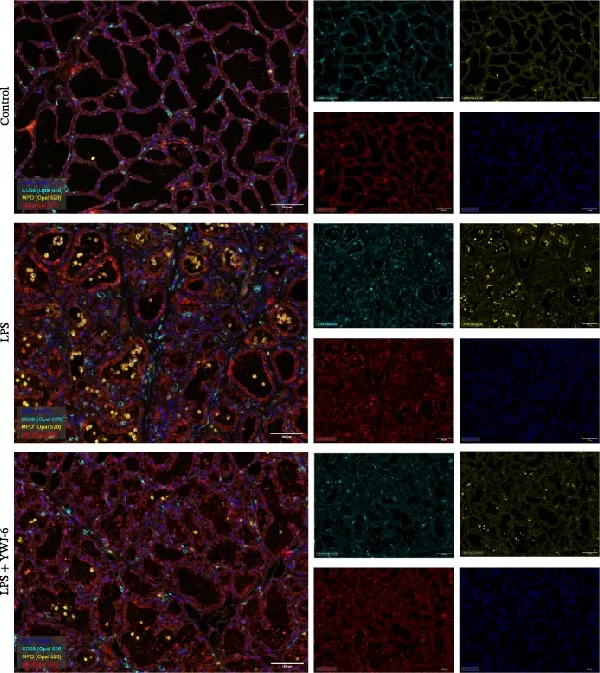
YWJ‐6 reduced inflammatory infiltration and inhibited NF‐κB p65 activation in LPS‐induced rat mastitis. Representative multiplex immunofluorescence images of mammary tissue sections from different groups. Sections were costained for CD68 (bright blue, marking macrophages), MPO (yellow, marking neutrophils), and p65 (red, indicating NF‐κB subunit). Nuclei were counterstained with DAPI (blue). 20x scale = 100 μm (*n* = 3).

The chemical structure of YWJ‐6 is presented in Figure 1A. All of the groups of mammary tissues were subjected to H&E staining and the MPO expression assay (Figure 1B,C). The Control group exhibited normal histological features, characterized by regularly arranged mammary lobules and intact ductal epithelia. In contrast, the model group exhibited extensive pathological changes, including disorganized mammary lobular and ductal structures, incomplete acinar architecture, and marked inflammatory cell infiltration within the acini. However, after YWJ‐6 treatment, the pathological manifestations of mammary tissues were markedly alleviated. MPO, an enzyme predominantly expressed in neutrophils, reflects the extent of neutrophil infiltration [27]. In tissues, MPO expression levels were significantly elevated following LPS stimulation, whereas in the YWJ‐6 group, this upregulation was suppressed. Meanwhile, the levels of IL‐1β, IL‐6, and TNF‐α were also significantly reduced following administration of YWJ‐6. (Figure 1D–J). Additionally, the correlation between the expression of CD68, MPO, and NF‐κB p65 in the breast tissue was simultaneously assessed using multiplex immunohistochemistry (Figure 2). Following LPS stimulation, elevated expression of CD68, MPO, and NF‐κB p65 proteins was observed in the tissue. These data confirmed the success of LPS in inducing mammary inflammation, the significant therapeutic efficacy of YWJ‐6 in rats, and suggested a potential association linking YWJ‐6 to the NF‐κB signaling pathway.

### 3.2. YWJ‐6 Protected the Integrity of the BMB

**Figure 3:**
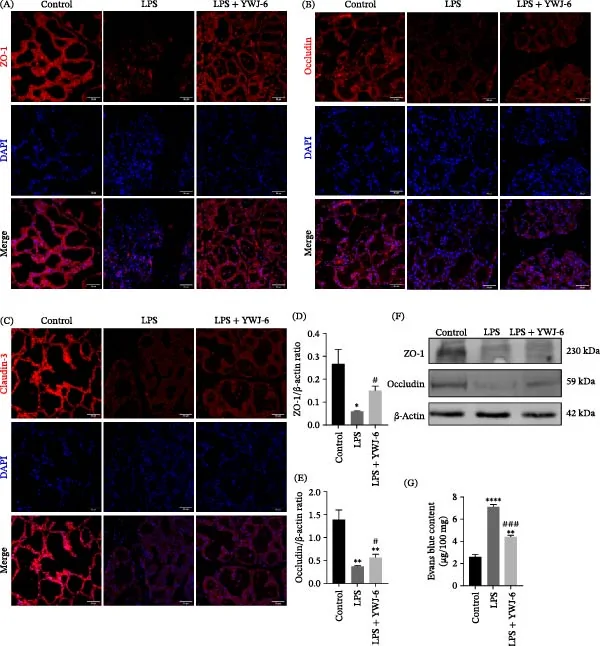
YWJ‐6 maintained the integrity of the blood–milk barrier by increasing the expression of TJ proteins. (A–C) Representative immunofluorescence staining images of ZO‐1, Occludin, and Claudin‐3 in mammary tissue. (D–F) Protein expression levels of ZO‐1 and Occludin in mammary tissue were determined by Western blot, with β‐actin used as the internal control. (G) Evans blue extravasation assay was performed to evaluate BMB permeability in vivo.  ^∗^ *p* < 0.05,  ^∗∗^ *p* < 0.01, and  ^∗∗∗∗^ *p* < 0.0001 versus Control group; #*p* < 0.05, ###*p* < 0.001 versus LPS group. Figure A–C scale = 50 μm (*n* = 3).

LPS impairs the integrity of the BMB, thereby exacerbating inflammatory responses [28]. To verify the protective effect of YWJ‐6 on BMB, our research examined the expression of tight junction (TJ) proteins in the tissue and vascular permeability in the tissue. Immunofluorescence results indicated that LPS stimulation reduced the expression of ZO‐1, Occludin, and Claudin‐3. However, YWJ‐6 treatment markedly restored their expression (Figure 3A–C). WB data specifically demonstrated that YWJ‐6 can attenuate the LPS‐induced downregulation of TJ proteins (Figure 3D–F). Consistently, the Evans blue extravasation assay showed that LPS significantly increased dye leakage into mammary tissue, indicating enhanced BMB permeability, whereas YWJ‐6 treatment markedly reduced this leakage (Figure 3G). Collectively, these findings indicate that YWJ‐6 preserves BMB function via maintaining the abundance of the TJ protein.

### 3.3. YWJ‐6 Alleviated Inflammatory Response in LPS‐Stimulated HC11 Cells

**Figure 4:**
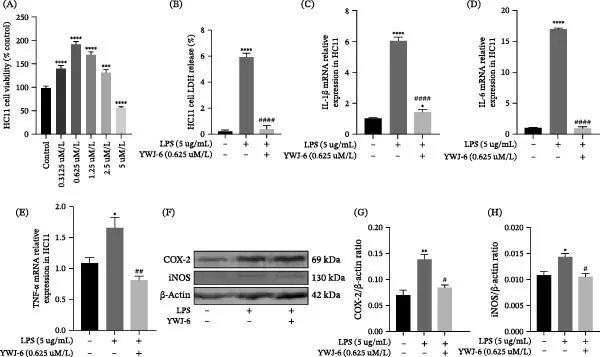
YWJ‐6 alleviates the inflammatory response in LPS‐stimulated HC11 cells. (A) The effect of different concentrations of YWJ‐6 on viability of HC11 cells was determined by the CCK‐8 assay (*n* = 6). (B) Effect of YWJ‐6 on LDH release levels in LPS‐stimulated HC11 cells (*n* = 4). (C–E) mRNA expression levels of IL‐β, IL‐6, and TNF‐α were measured using RT‐qPCR (*n* = 3). (F–H) Protein expression levels of COX‐2 and iNOS in HC11 cells were detected by Western blot, with β‐actin used as the internal control (*n* = 3).  ^∗^ *p* < 0.05,  ^∗∗^ *p* < 0.01,  ^∗∗∗^ *p* < 0.001, and  ^∗∗∗∗^ *p* < 0.0001 versus Control group; #*p* < 0.05, ##*p* < 0.01, and ####*p* < 0.0001 versus LPS group.

HC11 cells serve as an in vitro model system to investigate mammary epithelial cell function. As the primary functional units of the mammary gland, these cells can maintain the homeostasis of the mammary tissue [28]. Previous studies have demonstrated that LPS can activate inflammation in mammary epithelial cells, triggering proinflammatory cascades [29]. In our experimental design, inflammation was induced by stimulating HC11 cells with LPS for 18 h. Initial cytotoxicity was evaluated via the CCK‐8 assay, which revealed no significant cytotoxicity of YWJ‐6 at concentrations below 2.5 μmol/L (Figure 4A). To better mimic the physiological relevance of in vivo settings while considering the cytotoxic profile of YWJ‐6 in RAW264.7 cells, a safe concentration of 1.25 μmol/L was selected for subsequent experiments. The release of LDH can reflect the degree of cellular damage. Our researchers discovered through experiments that YWJ‐6 can effectively reduce the level of LDH release induced by LPS. Our results demonstrated that YWJ‐6 reduces the expression of proinflammatory mediators induced by LPS (Figure 4C–G). These findings led us to conclude that YWJ‐6 attenuates LPS‐triggered inflammatory responses in HC11 cells.

### 3.4. YWJ‐6 Alleviated Inflammatory Response in RAW264.7 Cells

**Figure 5:**
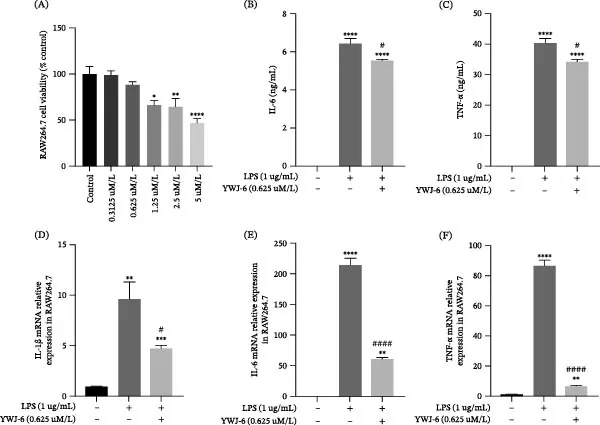
YWJ‐6 alleviates inflammatory response in LPS‐stimulated RAW264.7 cells. (A) The effect of different concentrations of YWJ‐6 on the viability of RAW264.7 cells was determined by the CCK‐8 assay (*n* = 4). Levels of (B) IL‐6 and (C) TNF‐α in the culture supernatants of RAW264.7 cells were measured using ELISA (*n* = 3). (D–F) mRNA expressions of IL‐β, IL‐6, and TNF‐α in RAW264.7 cells by RT‐qPCR (*n* = 3).  ^∗^ *p* < 0.05,  ^∗∗^ *p* < 0.01,  ^∗∗∗^ *p* < 0.001, and  ^∗∗∗∗^ *p* < 0.0001 versus Control group; #*p* < 0.05 and ####*p* < 0.0001 versus LPS group.

RAW264.7 cells are a key cell type mediating inflammatory responses. Upon LPS stimulation, these cells secrete inflammatory cytokines and trigger immune responses. Therefore, we assessed the impact of YWJ‐6 on mRNA expression and secretion stages of IL‐1β, IL‐6, and TNF‐α in RAW264.7 cells utilizing RT‐qPCR and ELISA (Figure 5). The data revealed that YWJ‐6 significantly lowered the mRNA transcription and release of these inflammatory mediators. These findings indicate that YWJ‐6 effectively suppressed the expression and discharge of inflamed cytokines in RAW264.7 cells stimulated by LPS.

### 3.5. YWJ‐6 Inhibited the Activation of the NF‐κB Signal Transduction Pathway in LPS‐Induced HC11 and RAW264.7 Cells

**Figure 6:**
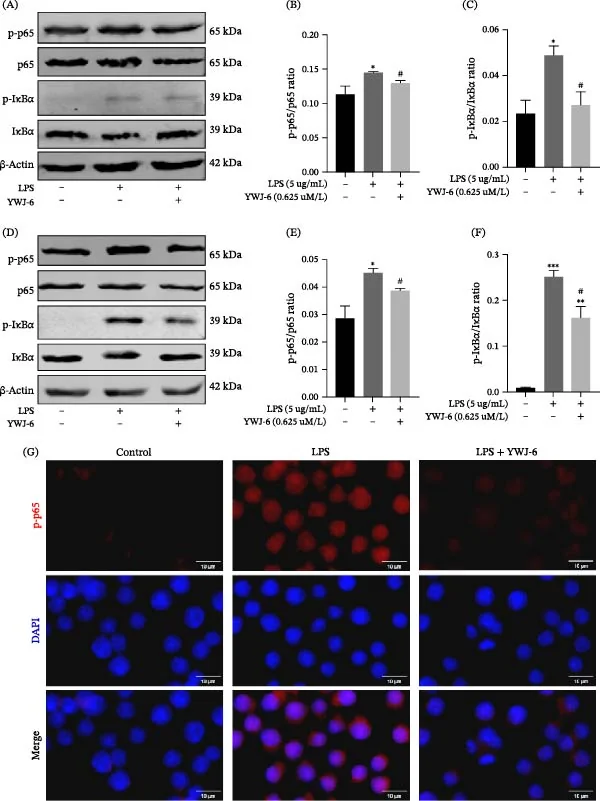
YWJ‐6 inhibits LPS‐induced NF‐κB pathway activation in HC11 and RAW264.7 cells. (A) Protein expression levels of NF‐κB p65, IκBα, p‐p65, and p‐IκBα in HC11 cells were determined by Western blot. (B, C) Western blot analysis of p‐p65 and p‐IκBα phosphorylation levels; the ratios of (B) p‐p65/p65 and (C) p‐IκBα/IκBα were quantified by densitometric analysis. (D) Protein expression levels of NF‐κB p65, IκBα, p‐p65, and p‐IκBα in RAW264.7 cells were determined by Western blot. (E, F) Western blot analysis of p‐p65 and p‐IκBα phosphorylation levels; the ratios of (E) p‐p65/p65 and (F) p‐IκBα/IκBα were quantified by densitometric analysis. (G) Representative immunofluorescence images of p‐p65 localization and quantitative analysis (nuclei stained with DAPI [blue]; p‐p65 stained with phospho‐anti‐p65 antibody [red]). Figure G scale = 10 μm.  ^∗^ *p* < 0.05,  ^∗∗^ *p* < 0.01, and  ^∗∗∗^ *p* < 0.001 versus Control group; #*p* < 0.05 versus LPS group (*n* = 3).

The NF‐κB signaling pathway takes part in the initiation and progression of inflammation and plays a crucial role in mediating the production of proinflammatory cytokines [30]. Prior investigations have demonstrated that thiourea derivatives elicit anti‐inflammatory effects by altering the NF‐κB signaling pathway [17, 31]. Our multiplex immunofluorescence staining results further corroborated the participation of the NF‐κB subunit in the anti‐inflammatory mechanisms of YWJ‐6 in mammary tissue. To further verify the modulatory effects of YWJ‐6 on this pathway, we performed targeted quantification of key NF‐κB pathway proteins. Western blot analysis confirmed that LPS stimulation increased the proportion of phosphorylated NF‐κB p65 and IκBα in both HC11 and RAW264.7 cells, whereas YWJ‐6 treatment significantly abrogated this LPS‐induced phosphorylation (Figure 6A–F). Immunofluorescence staining consistently demonstrated that the LPS + YWJ‐6 group had lower phospho‐NF‐κB p65 expression in RAW264.7 cells compared to that of the LPS group. (Figure 6G).

## 4. Discussion

**Figure 7:**
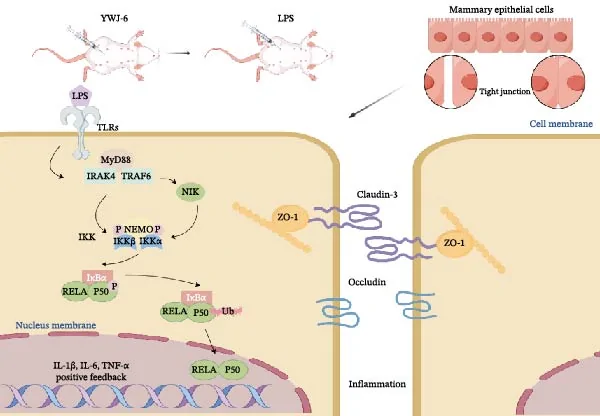
The mechanism of YWJ‐6 in alleviating mastitis and maintaining the blood–milk barrier.

Mastitis is a prevalent clinical disease affecting lactational women, with bacterial‐induced lactational mastitis being the most common form—typically caused by milk stasis and secondary infection [32]. The pathological manifestations of mastitis include increased blood flow, swelling, and damage to the mammary alveolar structure, accompanied by inflammatory cell infiltration [33]. Although antibiotic therapy remains the current standard of care, its clinical application is associated with various side effects [34]. Growing evidence suggests that inflammation damages the BMB, and restoring the damaged BMB is essential for reducing mastitis symptoms [35]. *Escherichia coli* is widely accepted as one of the primary causative agents of mastitis [36], and its LPS component is a key inducer of inflammatory cascades and BMB dysfunction. The present study demonstrates that YWJ‐6 exerts dual protective effects against LPS‐induced mastitis by modulating inflammatory responses and preserving BMB integrity. To evaluate these dual effects, we employed two complementary cell lines representing the key cellular players in mastitis pathogenesis. HC11 mammary epithelial cells, which constitute the structural foundation of the BMB and serve as the primary targets of inflammatory injury, were used to assess the barrier‐protective effects of YWJ‐6 on TJ proteins. RAW264.7 macrophages, which act as the first line of defense against bacterial invasion and orchestrate the inflammatory cascade through NF‐κB activation and proinflammatory cytokine production, were used to evaluate the anti‐inflammatory activity of YWJ‐6. By utilizing both cell types, we aimed to recapitulate the complex cellular interactions occurring in the mammary gland microenvironment, thereby providing a more comprehensive evaluation of YWJ‐6’s therapeutic potential. Additionally, multilayered cross‐validation was achieved via histopathological (H&E, IHC, and mIHC), biochemical (MPO, ELISA), molecular (RT‐qPCR, Western blot) methods, and Evans blue extravasation as a direct functional indicator of BMB permeability. Together, these methods formed a complete evidence chain, ensuring the reliability of our conclusions (Figure 7).

Thiosemicarbazone derivatives are a class of chelators with pivotal roles in inflammation regulation, and previous studies have reported their varied biological functions, such as antibacterial, antimalarial, antitumor, and anti‐inflammatory influence [13, 14, 31, 34]. For instance, the prototypical compound Dp44mT exerts potent antitumor activity in vitro and significantly suppresses macrophage secretion of TNF‐α and IL‐6 [17], demonstrating the anti‐inflammatory potential of this compound class. However, few studies have explored their therapeutic potential in mastitis, especially their effects on the BMB integrity. In our model, we observed elevated proinflammatory cytokines alongside reduced TJ protein expression—key structural components of the BMB. Notably, YWJ‐6 treatment markedly attenuated LPS‐induced inflammation and partially restored TJ protein levels, highlighting its advantage over conventional anti‐inflammatory agents that often target only a single pathway.

The BMB, with mammary epithelial cells as its structural core, relies on TJs (composed of Occludin, claudin family proteins, and cytoplasmic scaffold protein ZO‐1) to maintain selective permeability [37, 38]. Beyond regulating nutrient transport between blood and milk, the BMB defends against pathogen/toxin invasion into milk [39, 40]. A hallmark of mastitis is massive immune cell influx into the mammary tissue, which disrupts BMB integrity and downregulates TJ protein expression [41]. This damage further facilitates bacterial invasion, exacerbating tissue injury and inflammatory amplification. Our data indicate that YWJ‐6 partially reverses LPS‐induced suppression of TJ proteins, thereby protecting BMB integrity and subsequently mitigating inflammation—representing a novel mechanism for YWJ‐6 in mastitis treatment.

The NF‐κB signaling pathway is essential for the progression of acute and chronic inflammatory conditions, making the pathway a key therapeutic target for anti‐inflammation drugs. IκB proteins retain NF‐κB in the cytoplasm when inactive [42, 43]. Upon LPS binding to Toll‐like receptor 4 (TLR4), the IκB kinase (IKK) is activated, leading to IκBα phosphorylation and degradation [44, 45], releasing NF‐κB for nuclear translocation and transcription of inflammatory genes [38]. The results demonstrate that YWJ‐6 significantly suppresses LPS‐induced NF‐κB activation by inhibiting IκBα phosphorylation/degradation and downregulating NF‐κB‐dependent cytokines. Thus, YWJ‐6 inhibits NF‐κB to directly alleviate inflammation and indirectly preserve BMB integrity via TJ upregulation, forming a synergistic antimastitis effect.

A key advantage of YWJ‐6 over conventional antibiotics is its mechanism as a host‐directed therapeutic agent. Antibiotics face challenges including antimicrobial resistance and side effects, whereas YWJ‐6 suppresses excessive inflammation via NF‐κB inhibition and preserves BMB integrity—targeting pathological consequences rather than the pathogen itself. This positions YWJ‐6 as a promising candidate for antibiotic‐sparing mastitis management. The dual effects of YWJ‐6—NF‐κB inhibition and barrier protection.

Admittedly, several limitations should be acknowledged. First, we acknowledge that our conclusion regarding NF‐κB is limited to inhibition of pathway activation (as evidenced by reduced p65 and IκBα phosphorylation), as we did not perform nuclear–cytoplasmic fractionation to quantitatively assess p65 nuclear translocation. Second, the pharmacokinetics, tissue distribution, and safety of YWJ‐6 in animals remain unknown; PK/PD studies are needed for clinical translation. Third, the dual effects of YWJ‐6 may also extend beyond mastitis as NF‐κB hyperactivation and barrier dysfunction are common features of IBD and sepsis. In summary, YWJ‐6 plays a key protective role in LPS‐induced mastitis by inhibiting NF‐κB signaling and restoring the expression of TJ proteins to maintain the integrity of the BMB. These findings provide a scientific basis for the anti‐inflammatory effects of YWJ‐6 and offer new insights into the pharmacological treatment of lactational mastitis and other inflammatory diseases.

## Author Contributions

Wei Tao, Ying Liu, Jia Liang, and Ling Guo designed and supervised the study. Ling Guo and Yining Wang performed the research, data analyses, and basic studies. Wei Tao, Ying Liu, and Jia Liang were responsible for the study’s reviewing and editing the manuscript.

## Funding

This study was supported by the Natural Science Foundation of Liaoning Province (Grant 2024‐MS‐201) and the Liaoning Education Innovation Development Project (Grant LJ242510160002).

## Disclosure

Wei Tao and Ying Liu reviewed and gave their approval to the final manuscript.

## Ethics Statement

The animal experimental protocol was approved by the Institutional Animal Care and Use Ethics Committee of Jinzhou Medical University (Approval Number 240243‐5). All procedures were conducted in line with the relevant national and international protocols.

## Conflicts of Interest

The authors declare no conflicts of interest.

## Supporting Information

Additional supporting information can be found online in the Supporting Information section.

## Supporting information


**Supporting Information** Figure S1: ^13^C NMR spectrum (101 MHz, DMSO‐d_6_). Figure S2: ^1^H NMR spectrum (400 MHz, DMSO‐d_6_) of YWJ‐6. Figure S3: ^13^C NMR spectrum (101 MHz, DMSO‐d_6_) of YWJ‐6. Figure S4: Mass spectrum of YWJ‐6. Figure S5: Original, uncropped Western blot images. (A) Corresponds to Figure 3F (ZO‐1 in mammary tissue). (B) Corresponds to Figure 3F (Occludin in mammary tissue). (C) Corresponds to Figure 3F (β‐actin in mammary tissue). (D) Corresponds to Figure 4F (COX‐2+β‐actin in HC11). (E) Corresponds to Figure 4F (iNOS in HC11). (F) Corresponds to Figure 6A (p‐p65 in HC11). (G) Corresponds to Figure 6A (p65+β‐actin in HC11). (H) Corresponds to Figure 6A (p‐IκBα in HC11). (I) Corresponds to Figure 6A ((IκBα in HC11). (J) Corresponds to Figure 6D (p‐p65 in RAW264.7). (K) Corresponds to Figure 6D (p65+β‐actin in RAW264.7). (L) Corresponds to Figure 6D (p‐IκBα in RAW264.7). (M) Corresponds to Figure 6D (p‐IκBα in RAW264.7).

## Data Availability

All the raw data and code can be provided if requested.
