# Supplementary material for: Therapeutic Potential of Thiosemicarbazone Derivative in LPS‐Induced Mastitis: Dual Modulation of Inflammatory Response and Blood–Milk Barrier Integrity
Source: J Immunol Res. 2026 Sep 21;2026:7518397. doi: 10.1155/jimr/7518397 (PMC13592133; doi:10.1155/jimr/7518397)

Supplementary Information

Therapeutic Potential of Thiosemicarbazone Derivative in LPS-Induced Mastitis: Dual Modulation of Inflammatory Response and Blood-milk Barrier Integrity

Ling Guo[1, a, b#], Yining Wang [2, a, c#], Jia Liang [a, d], Ying Liu [a, e*], Wei Tao [c*]

[a] Institution of Life Science, Jinzhou Medical University, Jinzhou, China

[b] Department of Cardiovascular Surgery, Jinzhou Central Hospital, Jinzhou, China

[c] The First Affiliated Hospital of Jinzhou Medical University, Jinzhou, China

[d] Key Laboratory of Neurodegenerative Diseases of Liaoning Province and Department of Neurobiology, Jinzhou Medical University, Jinzhou, China

[e] Institution of Basic Medical Science, Jinzhou Medical University, Jinzhou, China

* Corresponding author:

Wei Tao, The First Affiliated Hospital of Jinzhou Medical University, Jinzhou, China, E-mail: tw198122@sina.com

Ying Liu, Institution of Life Science, Jinzhou Medical University, Jinzhou, China, E-mail: yingliu@jzmu.edu.cn

[#] The authors contribute equally to this work

Other author's email:

Ling Guo, Institution of Life Science, Jinzhou Medical University, Jinzhou, China, E-mail: 403919807@qq.com

Yining Wang, Institution of Life Science, Jinzhou Medical University, Jinzhou, China, E-mail: 547399848@qq.com

Jia Liang, Key Laboratory of Neurodegenerative Diseases of Liaoning Province and Department of Neurobiology, Jinzhou Medical University, Jinzhou, China, liangjia@jzmu.edu.cn

Supporting figures

Fig.S1. ^{13}C NMR spectrum (101 MHz, DMSO-d_6) of YWJ-6

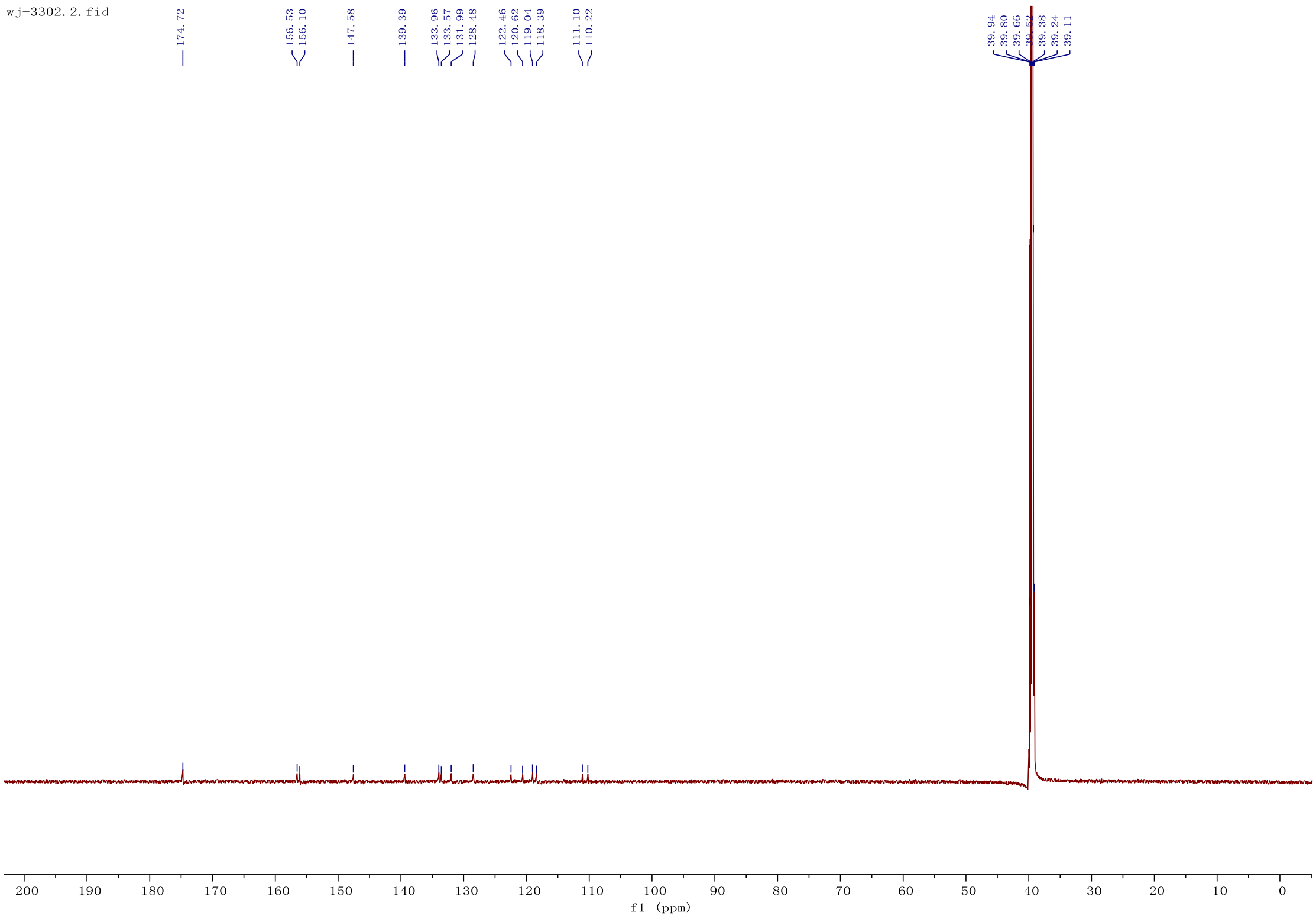

Fig.S2. ^{1}H NMR spectrum (400 MHz, DMSO-d_6) of YWJ-6

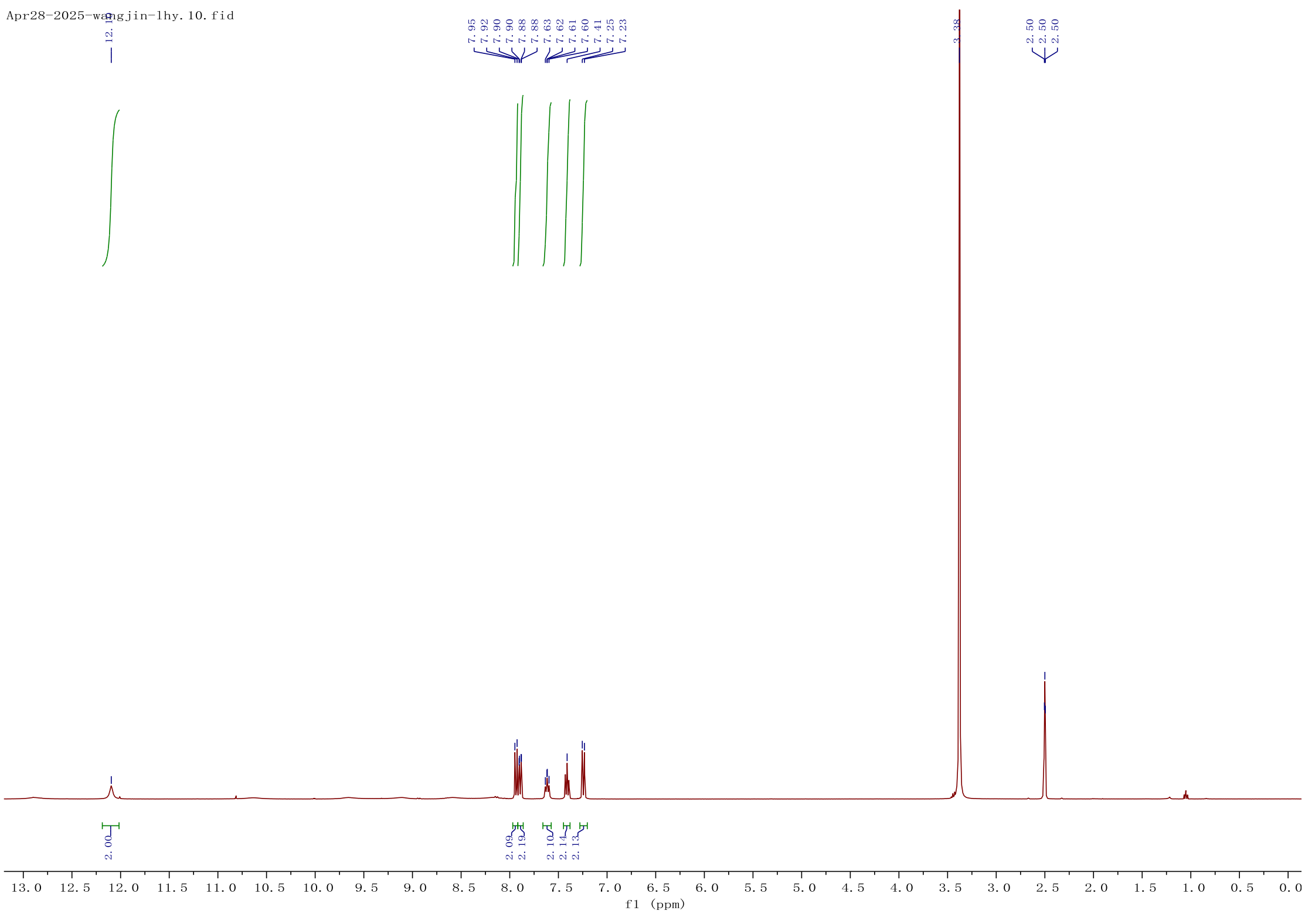

Fig.S3. ^{13}C NMR spectrum (101 MHz, DMSO-d_6) of YWJ-6

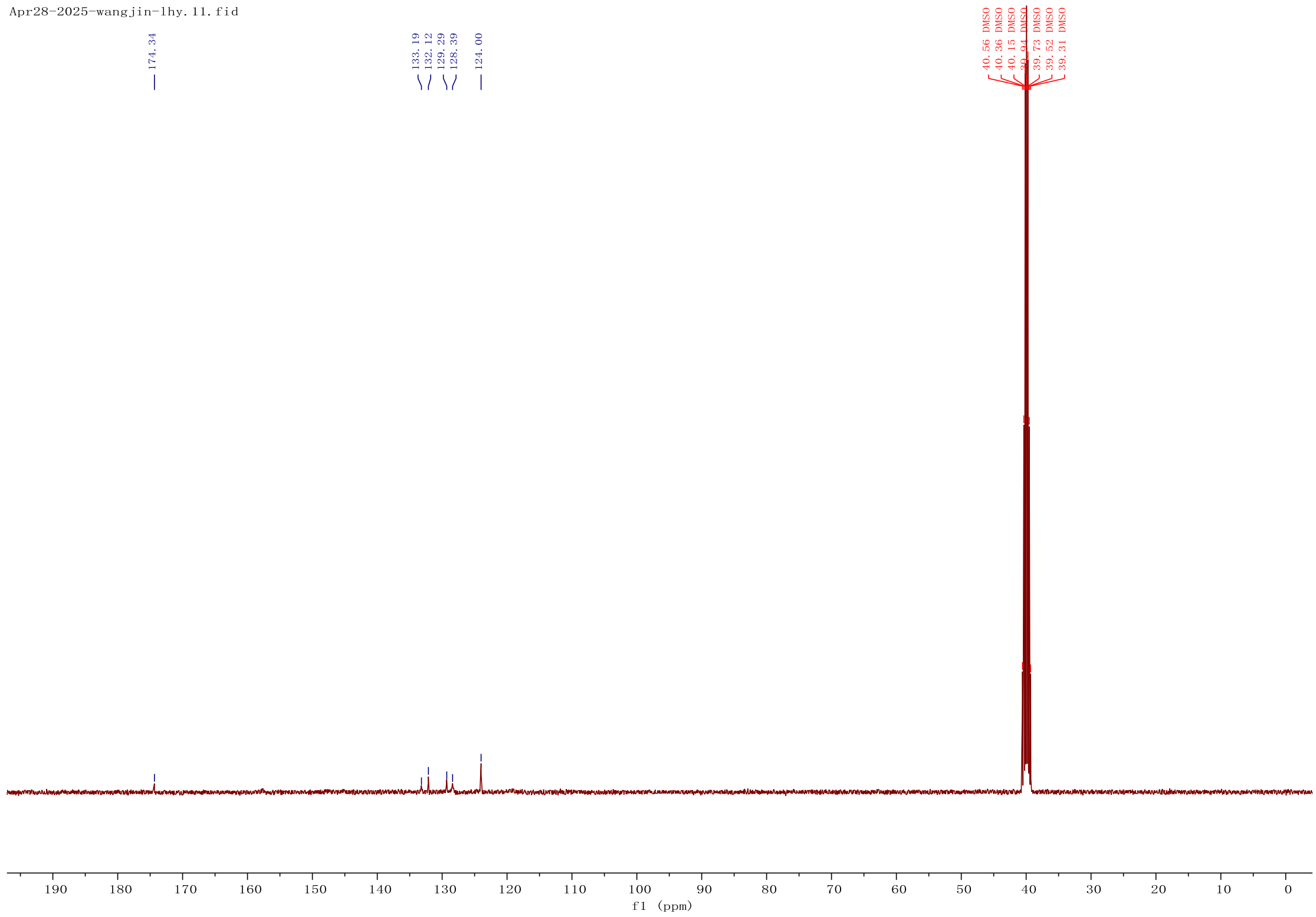

Fig.S4. Mass spectrum of YWJ-6

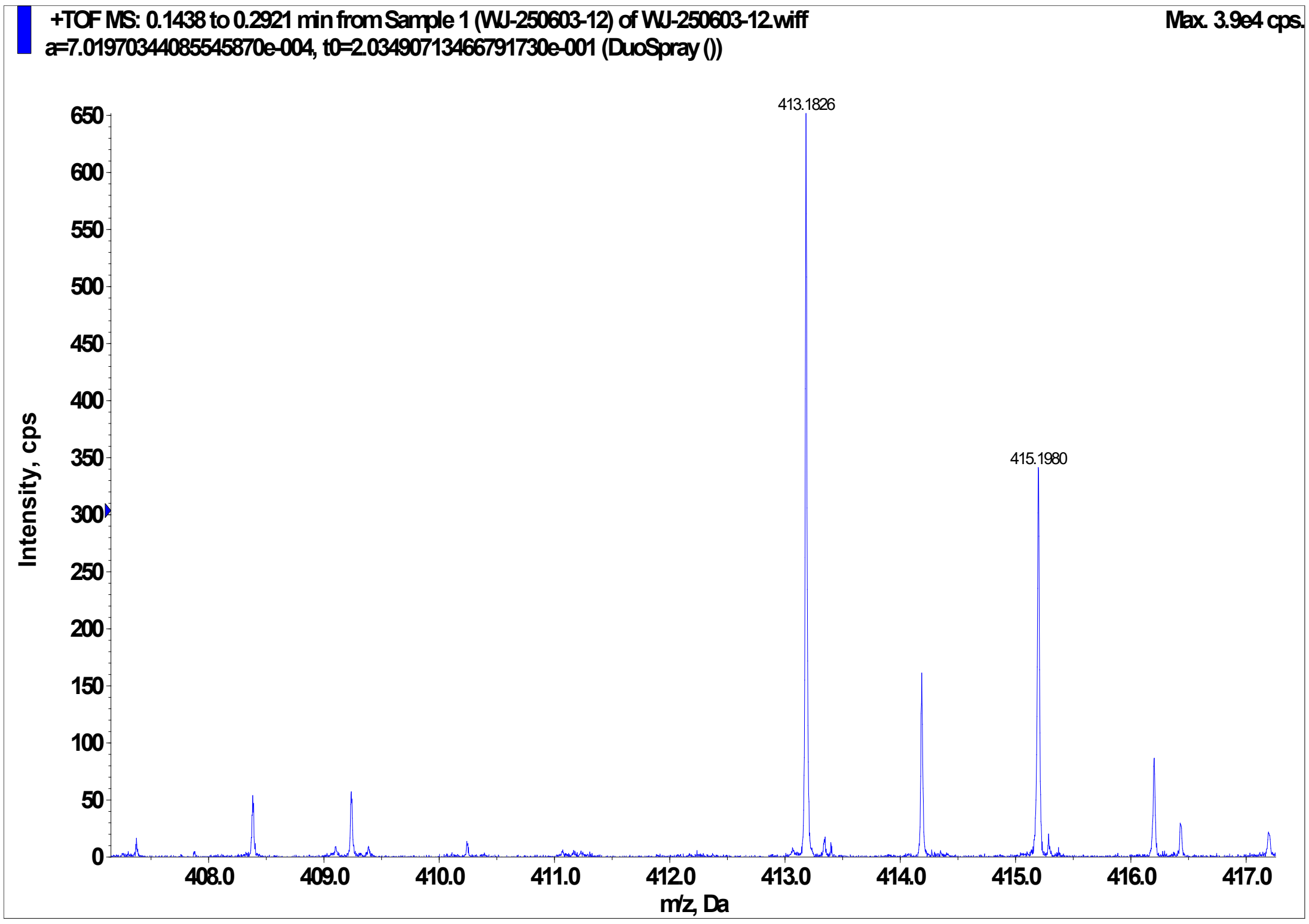

Fig.S5. Original, uncropped Western blot images

Fig.S5A. – Corresponds to Figure 3F (ZO-1 in mammary tissue)

Line 1: Control
Line 2: LPS
Line 3: LPS+YWJ-6
Replicate 2

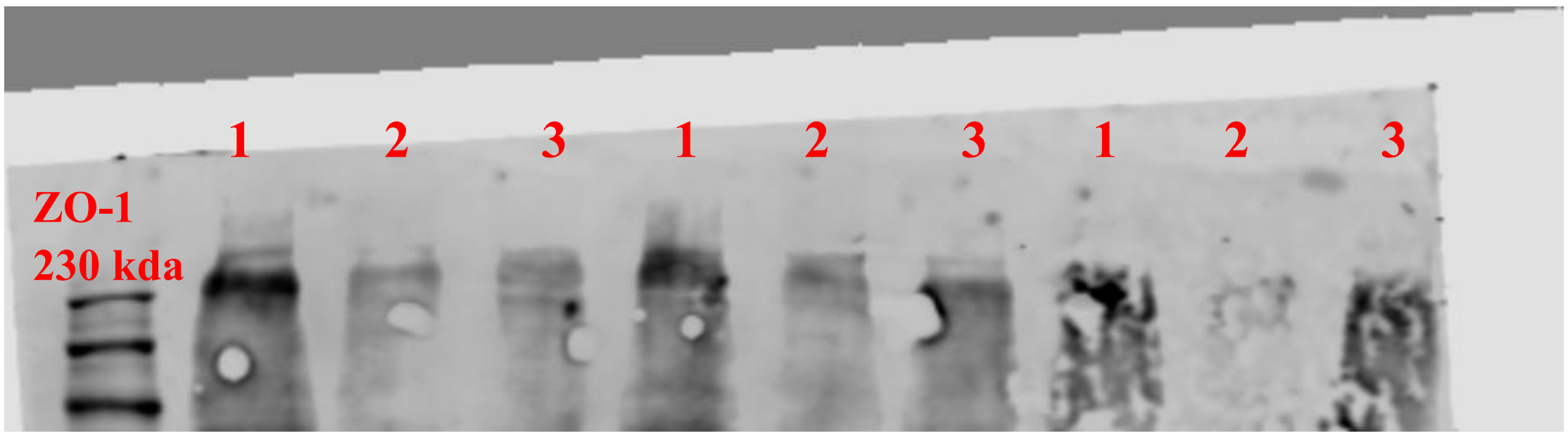

Fig.S5B. – Corresponds to Figure 3F (Occludin in mammary tissue)

Line 1: Control
Line 2: LPS
Line 3: LPS+YWJ-6
Replicate 2

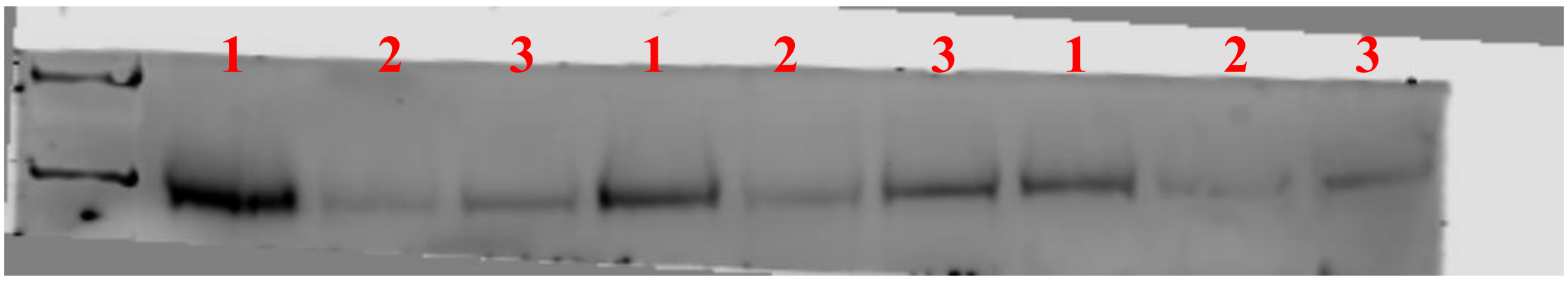

Fig.S5C. – Corresponds to Figure 3F (β-actin in mammary tissue)

Line 1: Control
Line 2: LPS
Line 3: LPS+YWJ-6
Replicate 2

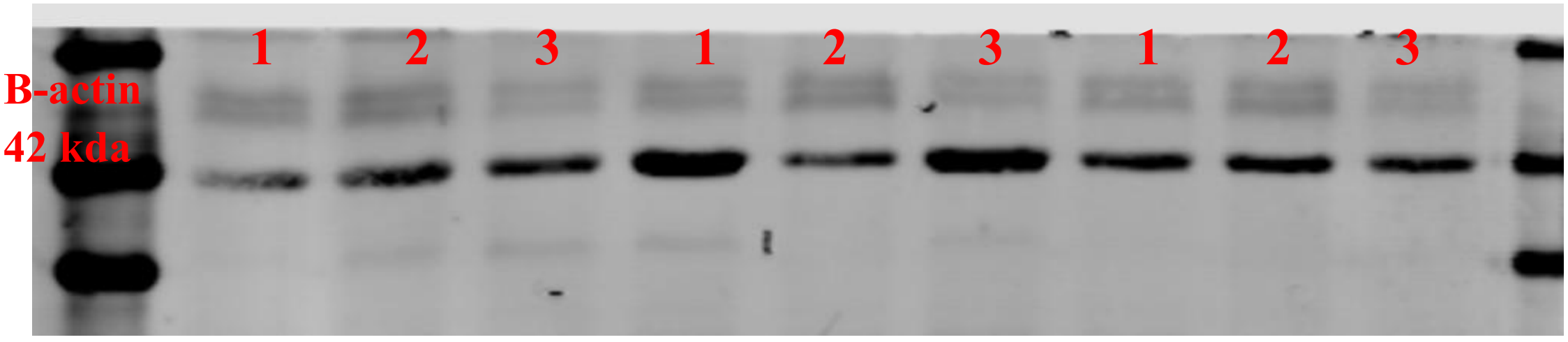

Note: β-actin and ZO-1 are on the same membrane; due to their significant difference in molecular weight, they were cropped during membrane transfer.

Fig.S5D. – Corresponds to Figure 4F (COX-2+β-actin in HC11)

Line 1: Control
Line 2: LPS
Line 3: LPS+YWJ-6
Replicate 2

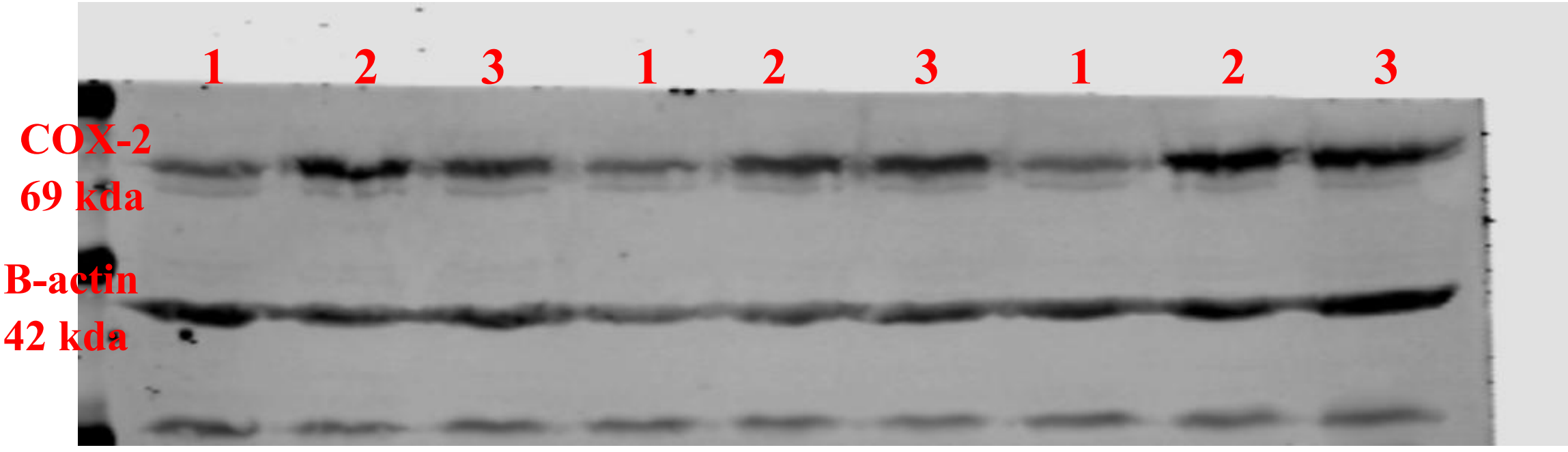

Fig.S5E. – Corresponds to Figure 4F (iNOS in HC11)

Line 1: Control
Line 2: LPS
Line 3: LPS+YWJ-6
Replicate 2

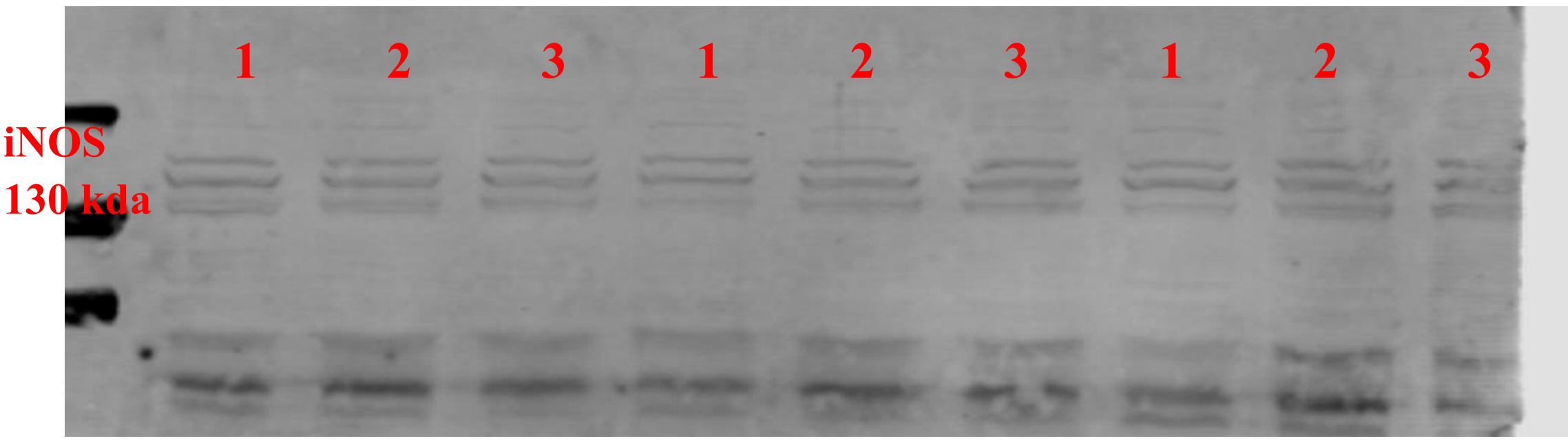

Fig.S5F. – Corresponds to Figure 6A (p-p65 in HC11)

Line 1: Control
Line 2: LPS
Line 3: LPS+YWJ-6
Replicate 2

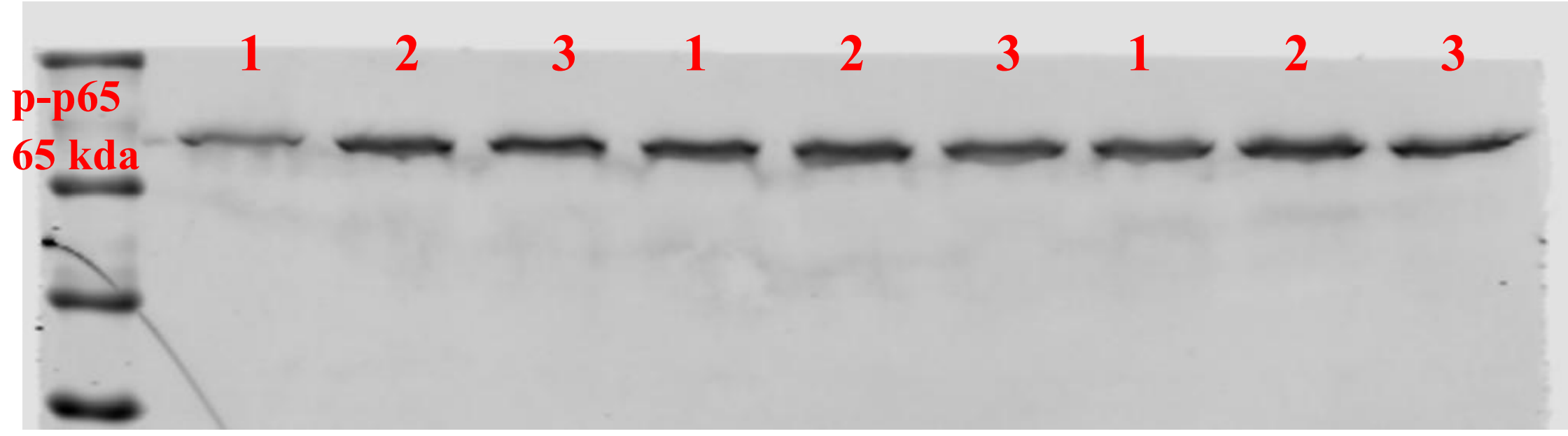

Fig.S5G. – Corresponds to Figure 6A (p65+β-actin in HC11)

Line 1: Control
Line 2: LPS
Line 3: LPS+YWJ-6
Replicate 2

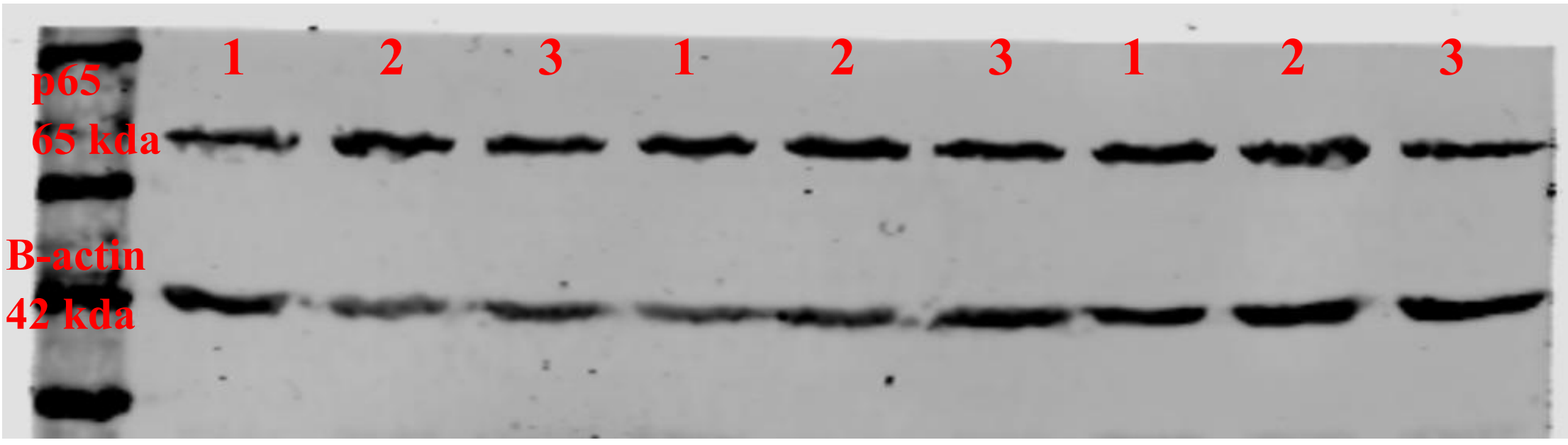

Fig.S5H. – Corresponds to Figure 6A (p-IκBα in HC11)

Line 1: Control
Line 2: LPS
Line 3: LPS+YWJ-6
Replicate 2

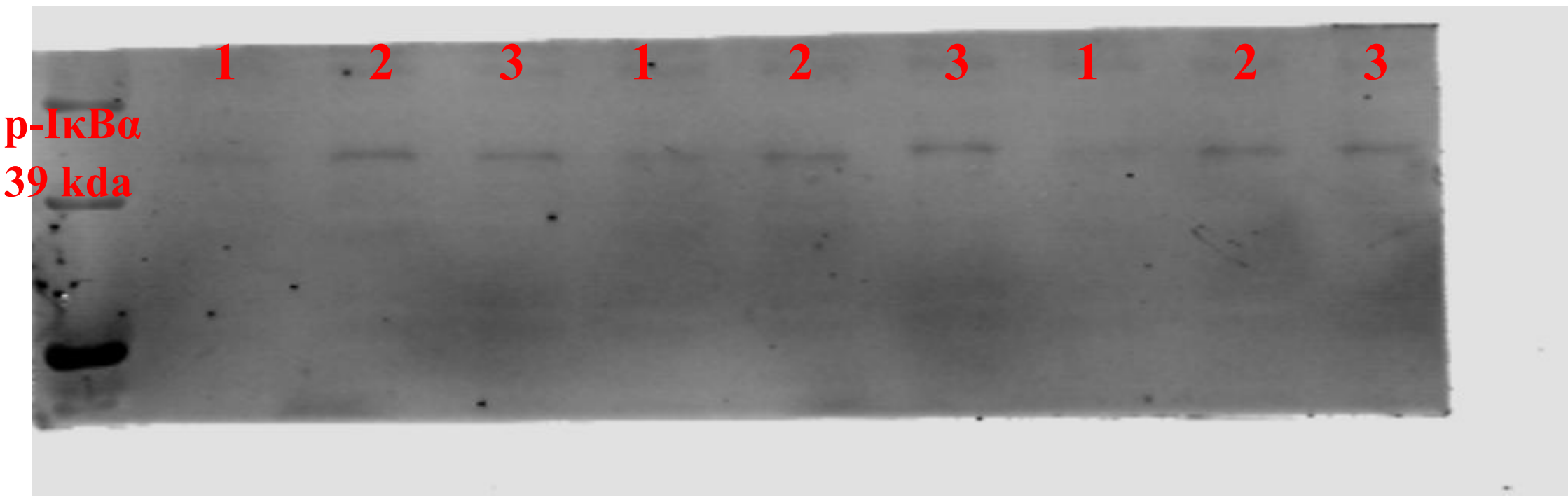

Fig.S5I. – Corresponds to Figure 6A ((IκBα in HC11)

Line 1: Control
Line 2: LPS
Line 3: LPS+YWJ-6
Replicate 2

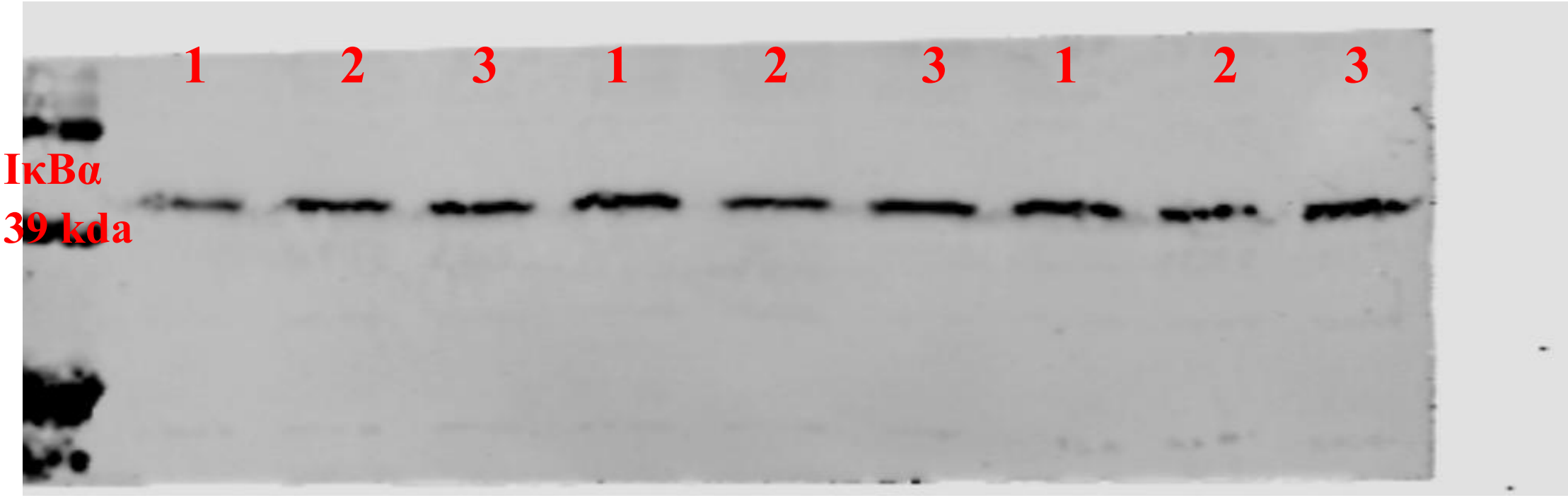

Fig.S5J. – Corresponds to Figure 6D (p-p65 in RAW264.7)

Line 1: Control
Line 2: LPS
Line 3: LPS+YWJ-6
Replicate 2

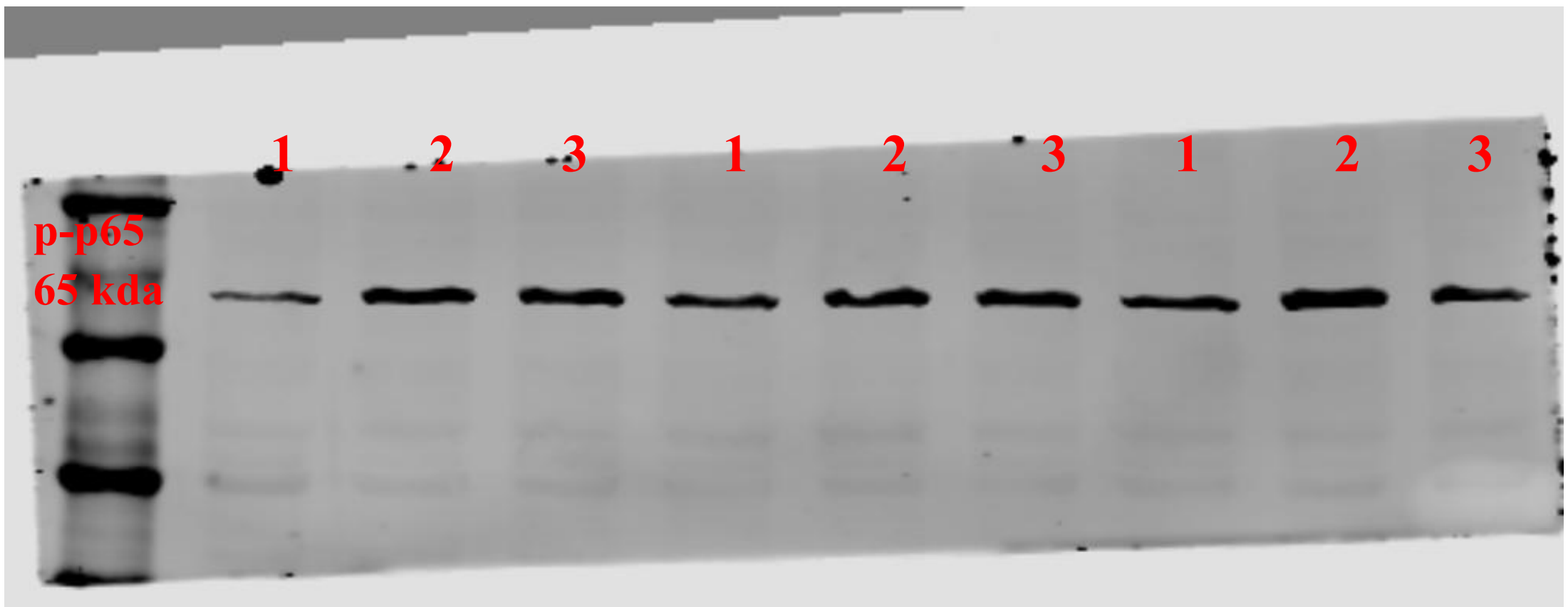

Fig.S5K. – Corresponds to Figure 6D (p65+β-actin in RAW264.7)

Line 1: Control
Line 2: LPS
Line 3: LPS+YWJ-6
Replicate 2

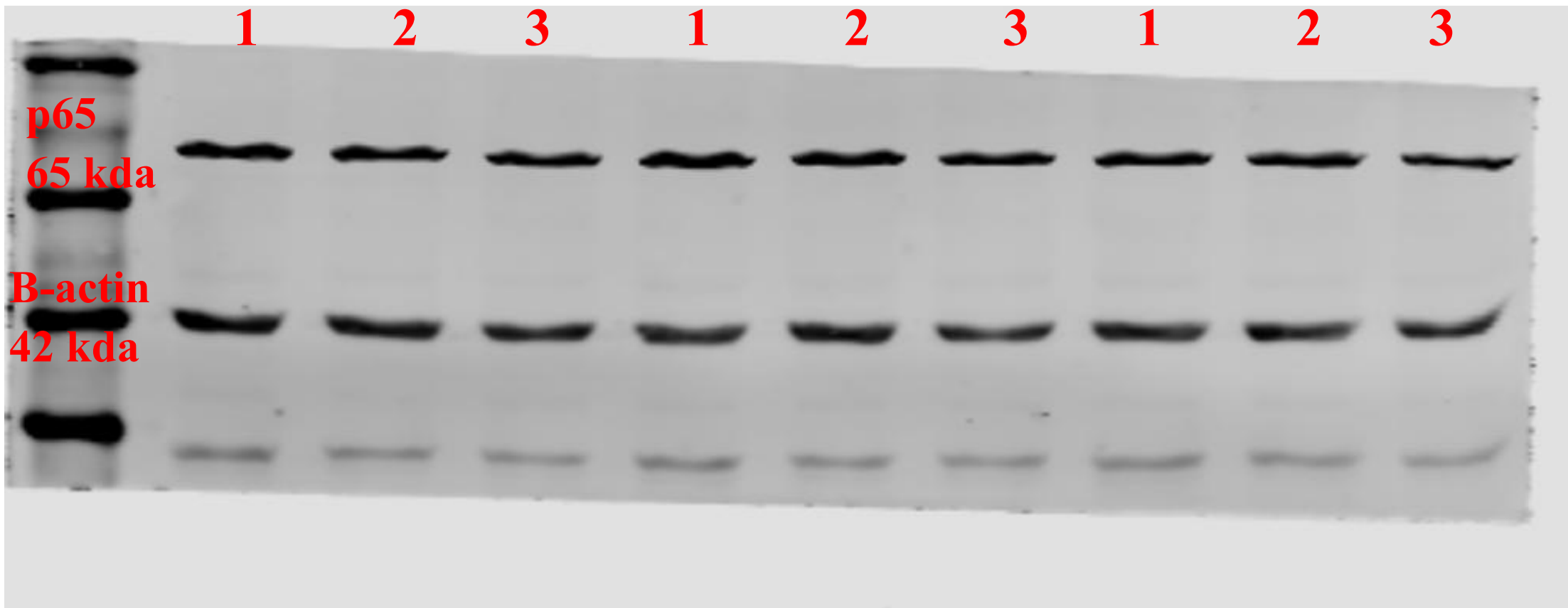

Fig.S5L. – Corresponds to Figure 6D (p-IκBα in RAW264.7)

Line 1: Control
Line 2: LPS
Line 3: LPS+YWJ-6
Replicate 2

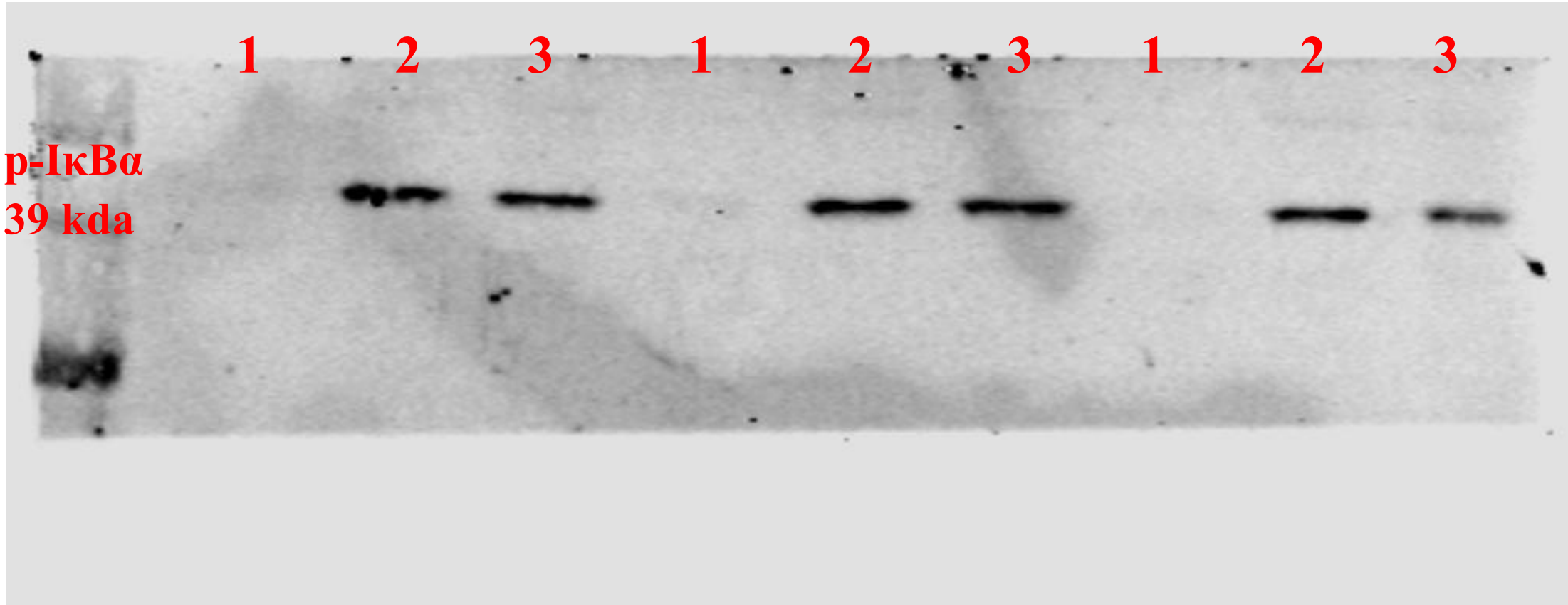

Fig.S5M. – Corresponds to Figure 6D (p-IκBα in RAW264.7)

Line 1: Control
Line 2: LPS
Line 3: LPS+YWJ-6
Replicate 2

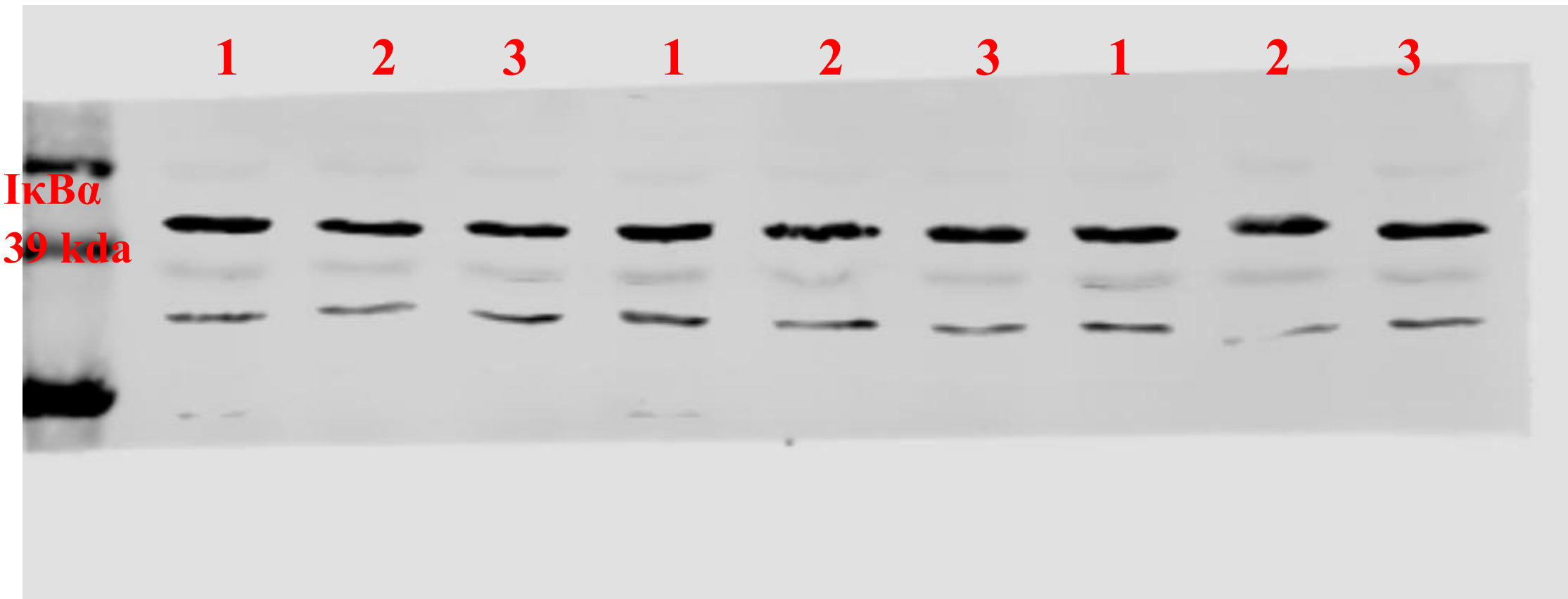

Supplement: Supplementary file 1 [file JIMR-2026-7518397-s001.pdf]
